# Impact of Artificial Intelligence on Periodontology: A Review

**DOI:** 10.7759/cureus.81162

**Published:** 2025-03-25

**Authors:** Maitri S Patel, Santosh Kumar, Bhavin Patel, Shirishkumar N Patel, Gaurav A Girdhar, Hiren H Patadiya, Tanvi Hirani, Mainul Haque

**Affiliations:** 1 Department of Periodontology and Implantology, Karnavati School of Dentistry, Karnavati University, Gandhinagar, IND; 2 Department of Dentistry, My Dental Southbridge PLLC, Southbridge, USA; 3 Department of Pharmacology and Therapeutics, National Defence University of Malaysia, Kuala Lumpur, MYS; 4 Department of Research, Karnavati School of Dentistry, Karnavati University, Gandhinagar, IND

**Keywords:** artificial intelligence, computer program, endodontics, exert influence, gum infection and disease, knowledge engineering, operating system, periodontology, tooth-related disease

## Abstract

Artificial intelligence (AI) is changing each of the healthcare fields, including periodontology, through the improvement of every diagnosis, treatment plan, and the handling of all patients. AI-driven technologies such as machine learning, deep learning, and computer vision are incorporated into radiographic analysis, automated disease detection, and prognosis prediction. These improvements effectively enable the early detection of periodontal diseases and efficient classification of disease severity. In addition, they allow for specially personalized treatment approaches. AI makes automated periodontal charting, virtual patient monitoring, and decision support systems easier, which improve clinical outcomes and patient care. Despite its immense potential, many substantial difficulties remain, such as data privacy, algorithm reliability, and the meaningful need for clinical validation. This review indicates the revolutionary function of AI in many current dental works and explores all present uses, advantages, limits, and possibilities in periodontology.

## Introduction and background

Periodontitis is a disease driven by microbial interactions and host-mediated inflammation, destroying periodontal attachment. Its pathophysiology involves key molecular pathways that trigger host-derived proteinases, contributing to the breakdown of marginal periodontal ligament fibers, the downward migration of the junctional epithelium, and the progression of bacterial biofilm along the root surface [1]. Clinicians often find it challenging to identify and diagnose periodontitis accurately [2]. The best practice involves assessing soft tissues using a calibrated probe [3] and evaluating hard tissues through radiographic imaging [4]. However, these techniques exhibit low inter- and intra-operator reliability due to inconsistencies in probing pressure and variations in radiographic angulation [2]. The diagnosis of periodontitis poses a challenge due to the intricate interplay of predisposing factors, which are difficult for clinicians and researchers to grasp fully. Given this complexity, AI is a valuable tool for analyzing these factors and enhancing understanding of the disease's diagnosis and etiology.

Problem statement of this paper

Periodontal diseases represent a significant global health issue, impacting millions and leading to both tooth loss and systemic health complications [5]. Conventional diagnostic and therapeutic strategies predominantly depend on clinical acumen, which may be inherently subjective and labor-intensive. The emergence of artificial intelligence (AI) presents a promising avenue to improve diagnostic precision, treatment planning, and patient outcomes within the realm of periodontology. Nevertheless, there remain substantial challenges in incorporating AI into clinical practice, encompassing issues related to data integrity, model dependability, and ethical implications. This review investigates the impacts of AI in periodontology, examining its prospective advantages, constraints, and future applications aimed at enhancing periodontal care [6].

Objectives of this narrative review paper

This review explores the transformative role of AI in periodontology, focusing on its applications in diagnosis, treatment planning, prognosis prediction, and patient management. It highlights AI advancements in periodontal disease detection, enhancing diagnostic accuracy and reducing subjectivity. The paper evaluates AI-based tools in treatment planning and their potential for predicting outcomes. It also examines challenges and limitations, including ethical concerns, data quality, and technological barriers. Additionally, it provides insights into the prospects of AI in periodontal care. This review offers a comprehensive understanding of AI's impact on clinical practice in periodontology.

## Review

Materials and methods

**Figure 1:**
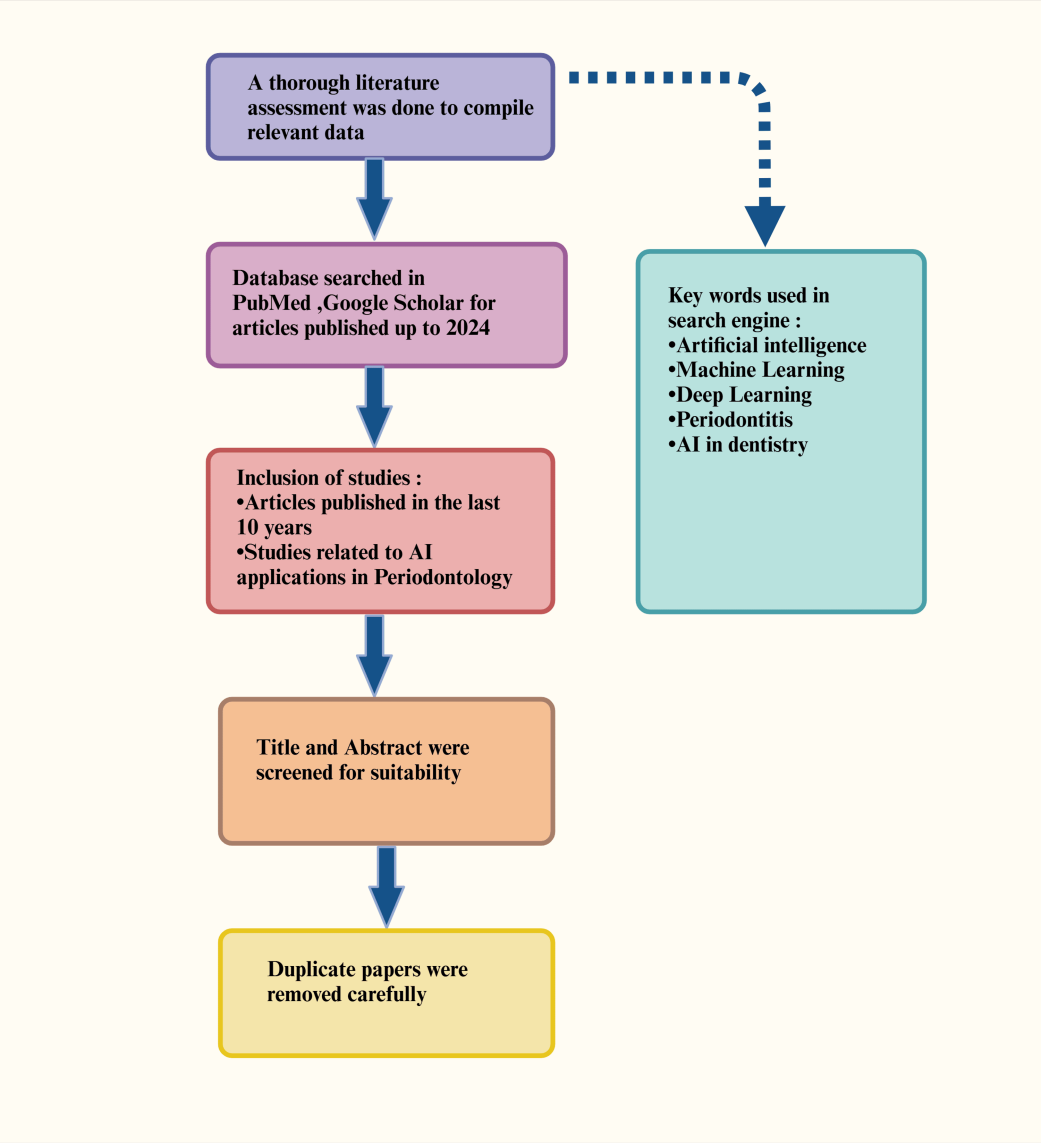
Diagram showing the methodology of the study. Note: This figure was drawn using the premium version of BioRender [7] (https://BioRender.com/), accessed on March 2, 2025, which has the agreement license number DE27Z6PHTO. Illustration credit: Maitri Patel

This review paper explores the role of AI in periodontology. These were all examined through a thorough literature assessment to compile relevant data. Databases such as PubMed, Scopus, Web of Science, and Google Scholar were used to identify relevant studies on AI applications in periodontology. The search included publications from 2009 to 2024 (15 years) with keywords such as "Artificial Intelligence in Periodontology" AND "Machine Learning in Dental Research". The inclusion criteria included studies that were peer-reviewed, written in English, and focused on AI, machine learning (ML), or deep learning (DL) applications in periodontal diagnosis, prognosis, or treatment. Eligible studies included original research, systematic reviews, and meta-analyses, while non-peer-reviewed articles, opinion pieces, and duplicates were excluded (Figure 1).

Review of literature

Definition of Artificial Intelligence

**Figure 2:**
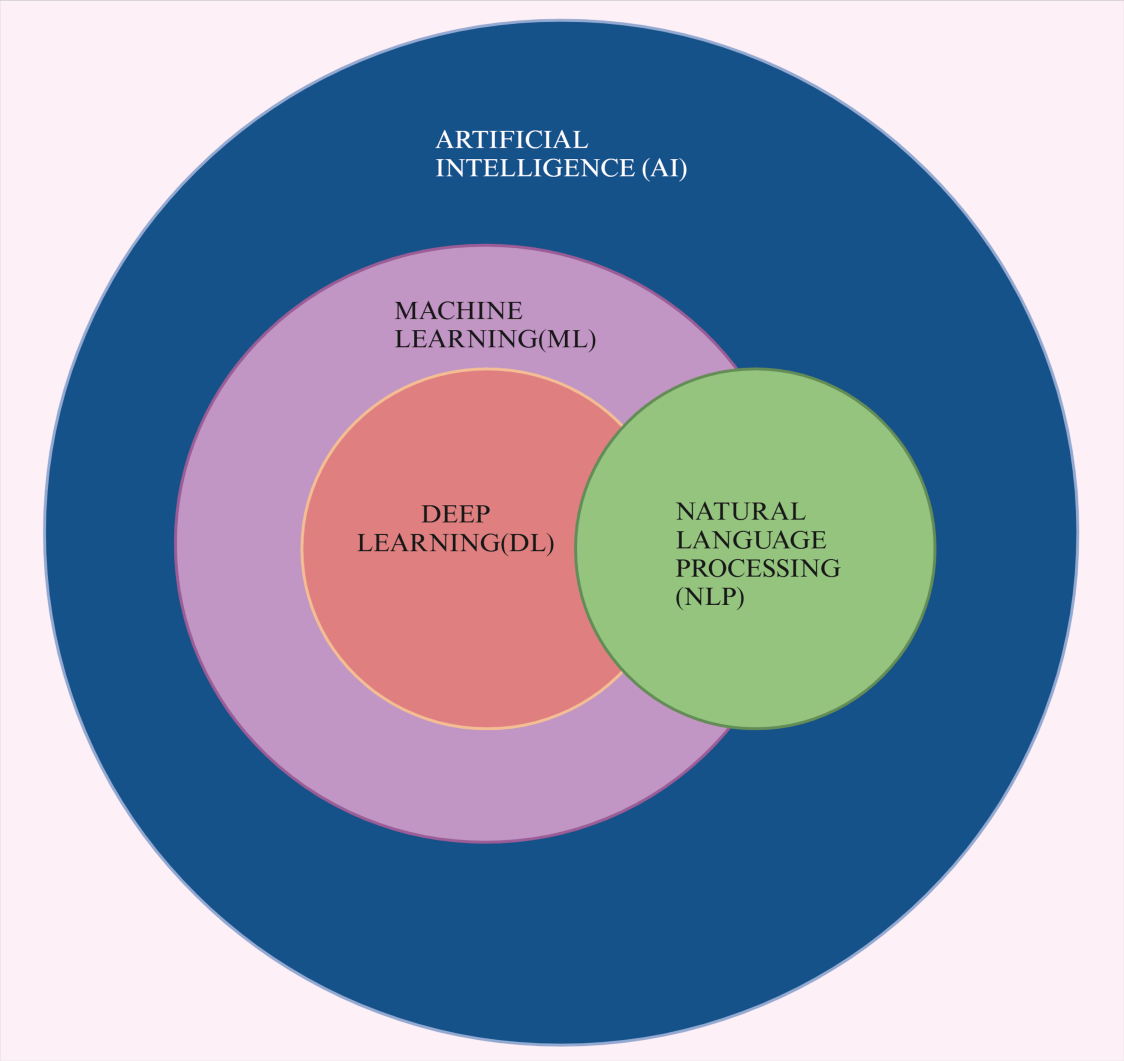
Diagram showing various branches of artificial intelligence. Note: This figure was drawn using the premium version of BioRender [7] (https://BioRender.com/), accessed on February 19, 2025), which has the agreement license number KD27XJCYL8. Illustration credit: Maitri Patel

AI constitutes a swiftly advancing domain within computer science, intending to develop machines capable of executing tasks traditionally necessitating human cognitive functions [8]. This field integrates a variety of methodologies, including ML, DL, and natural language processing (NLP). Large language models use deep knowledge and vast data to process and generate text (Figure 2). They support various NLP tasks, including translation, summarization, and sentiment analysis. As a branch of AI, NLP enables computers to understand and produce human language. AI has evolved from rule-based systems to advanced ML and DL methods [9].

Artificial Intelligence in Dentistry

**Figure 3:**
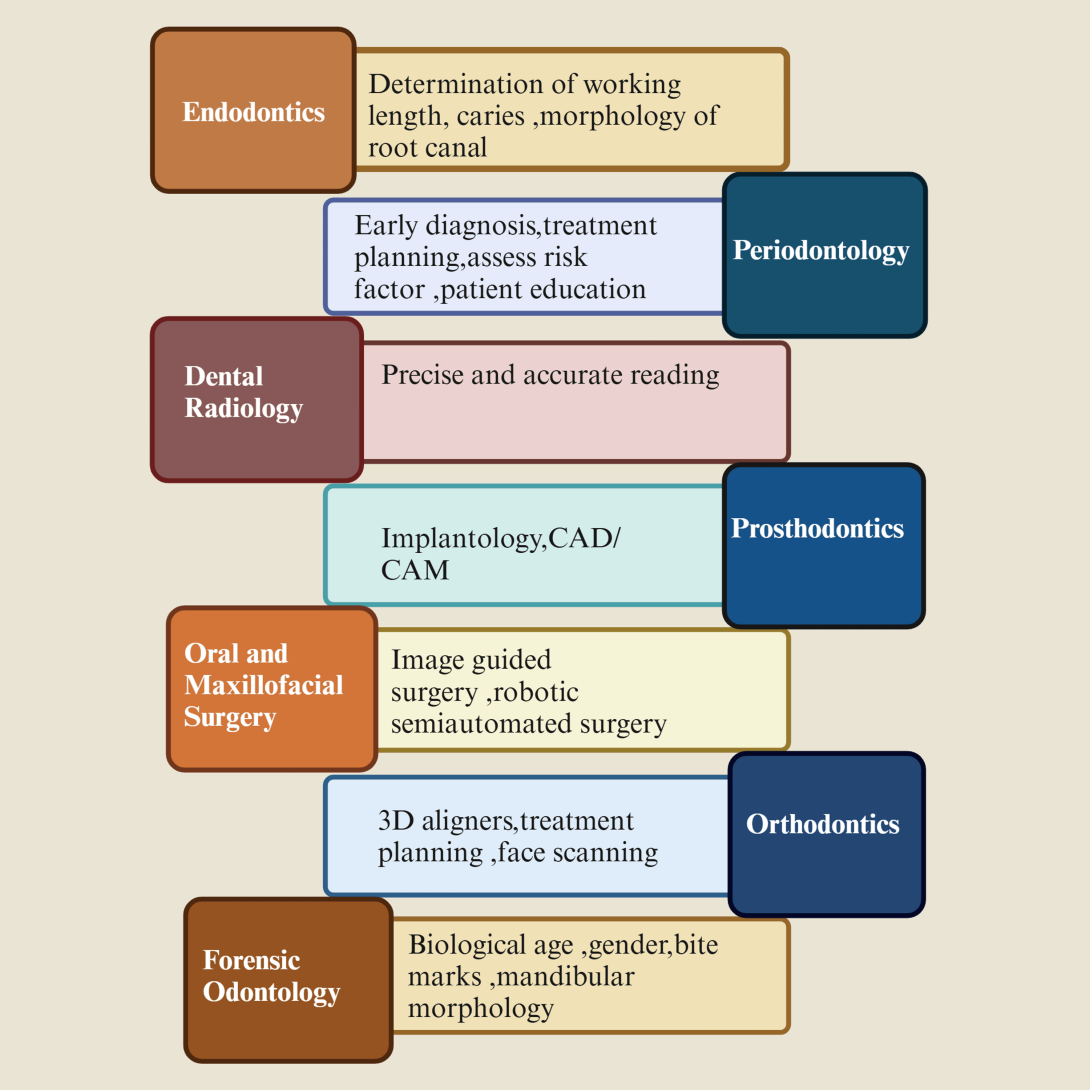
Diagram showing artificial intelligence in dentistry. Note: This figure was drawn using the premium version of BioRender [7] (https://BioRender.com/), accessed on February 20, 2025, which has the agreement license number VQ27XQJXQN. Illustration credit: Maitri Patel CAD, computer-aided design; CAM, computer-aided manufacturing

AI is rapidly advancing in dentistry, enhancing precision, reducing errors, and optimizing workflows to improve patient care (Figure 3). AI can perform various tasks within a dental clinic, including scheduling appointments and assisting in clinical diagnosis and treatment planning. AI improves efficiency, safety, and medical research by enabling personalized, predictive, and preventive dentistry, ultimately promoting sustainability and better healthcare outcomes [10]. AI has been utilized to enhance imaging analysis in dental radiology. A recent study showcased the potential of AI in endodontics to identify interproximal caries by analyzing a series of bitewing radiographs [11]. AI is employed in orthodontics to formulate treatment strategies and anticipate therapeutic outcomes, encompassing the simulation of facial aesthetic alterations before and after the intervention. In oral and maxillofacial pathology, AI has predominantly been investigated for its efficacy in identifying neoplasms and malignancies utilizing radiographic imagery and microscopic and ultrasonographic images. It also aids in identifying abnormalities in radiographs, including oral nerves, tongue muscles, and salivary glands [12]. Computer-aided design and manufacturing (CAD/CAM) technology is increasingly used in prosthetic dentistry, with AI integration improving chairside efficiency. The system streamlines design and manufacturing, allowing for milling or printing based on patient preferences. It creates 2D and 3D models fabricated using numerically controlled processes (Figure 3) [13].

Artificial Intelligence in Periodontology

Early diagnosis and risk assessment: Research has investigated AI algorithms for identifying periodontal diseases through clinical images, including intraoral and radiographic images. Krois et al. [14] formulated a deep-learning model to recognize periodontal bone loss, whereas Balaei et al. [15] employed convolutional neural networks (CNNs) to identify gingival bleeding. These investigations underscore the potential of AI in facilitating early diagnosis. Assessing the risk of progression in periodontal disease is crucial for developing effective treatment strategies and preventive interventions. Furthermore, AI can evaluate risk factors such as demographic information, medical history, and clinical data to forecast the progression of periodontal disease and inform treatment planning [16].

Automated radiographic analysis: Profound learning AI algorithms have demonstrated significant potential in analyzing radiographic images. They can autonomously identify early signs of periodontitis, such as bone loss and periodontal pocket formation, improving diagnostic accuracy while ensuring consistency and objectivity [17]. For instance, AI-driven software can assess digital radiographs in real-time, automatically evaluating bone levels and alveolar defects, allowing clinicians to detect periodontitis earlier than traditional techniques [18].

Cone beam computed tomography analysis: AI can now interpret cone beam computed tomography (CBCT) data to provide detailed 3D images of periodontal structures [19]. ML models allow for the exact diagnosis of periodontal abnormalities and bone resorption, improving the study of complex anatomy. AI-assisted CBCT analysis, for instance, can evaluate furcation involvement, alveolar bone height, and attachment loss more precisely than manual evaluation [20].

Differentiation between aggressive and chronic periodontitis: Feres et al. conducted a study utilizing a support vector machine-based classifier to examine the subgingival microbiome. Their research aimed to determine whether 40 bacterial species could effectively distinguish between generalized aggressive periodontitis in younger adults and generalized chronic periodontitis [21].

Personalized treatment planning: Personalized treatment planning (PTP) is integral in augmenting therapeutic outcomes and enhancing patient satisfaction within the domain of periodontics [22]. AI-driven algorithms can analyze patient-specific data, encompassing periodontal evaluations, genetic indicators, and individual treatment preferences, thereby facilitating the formulation of customized treatment protocols. Allahverdi and Akcan proposed a decision-support framework that employs fuzzy logic and genetic algorithms to refine the planning of periodontal therapies. By amalgamating patient preferences and clinical directives, AI-enhanced instruments can significantly bolster treatment adherence and improve long-term oral health results [23].

Post-treatment monitoring and maintenance: AI monitors and maintains long-term periodontal health after treatment [24]. AI-driven digital platforms analyze patient data from follow-up visits to track tissue healing and detect early signs of disease recurrence [25]. By assessing changes in periodontal pockets, tissue health, and bone density, these systems alert clinicians to potential issues before they escalate. Wearable devices and AI-powered mobile apps further enhance post-treatment care by tracking oral hygiene habits, such as brushing frequency and technique and providing personalized feedback for improvement [26]. For example, AI-integrated apps can identify missed areas based on biofilm presence and suggest adjustments for better maintenance [27]. Additionally, AI supports patient adherence by sending reminders for check-ups, medication schedules, and oral care routines [28]. Virtual assistants offer instant guidance, addressing concerns and reinforcing prescribed protocols [29].

Personalized and AI-supported periodontal education: Periodontology training traditionally uses standardized models, which may lead to memorization rather than authentic clinical learning. AI-driven augmented reality (AR) and virtual reality (VR) simulators offer dynamic periodontal models to enhance education [30]. AR overlays digital data onto real-world views, while VR immerses students in a virtual environment, improving hands-on experience. Although AR/VR training is widely used in dentistry, its application in periodontology remains limited [31]. Simulators such as PerioSim provide haptic feedback for tactile training but lack precise pocket depth and furcation involvement measurements [32]. Recent innovations, such as Haptodont, address these gaps by improving accuracy in probing techniques. Advanced AR/VR simulators can expand to personalized training, adapting to individual learning styles and clinical scenarios and ultimately enhancing students' preparedness for real-world periodontal care [33].

AI-powered teledentistry and virtual dental consultations: AI integration in telemedicine and teledentistry has expanded access to remote periodontal care. AI-powered platforms enable virtual consultations, allowing patients to connect with periodontists, discuss concerns, and receive preliminary assessments without visiting a clinic [34]. These systems utilize AI-driven image analysis to examine oral health conditions through uploaded photos or live video, providing initial diagnoses and recommendations [35]. This technology bridges the gap between patients and specialists, particularly in underserved or remote areas, ensuring timely guidance and interventions [36].

**Figure 4:**
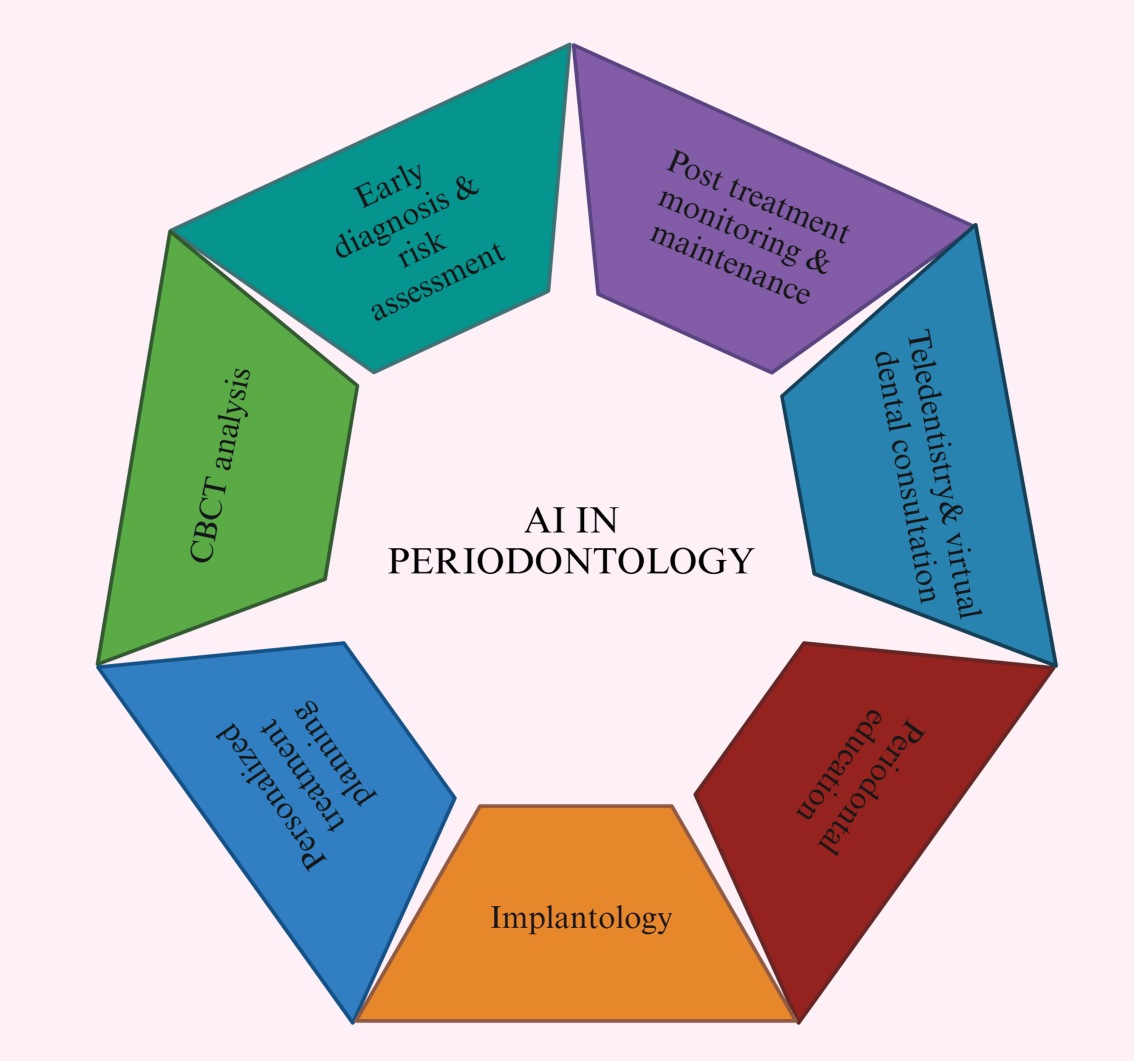
Diagram showing artificial intelligence in periodontology. Note: This figure was drawn using the premium version of BioRender [7] (https://BioRender.com/), accessed on March 2, 2025, which has the agreement license number NE27Z5RW4R. Illustration credit: Maitri Patel

Applications in implantology: A method for creating implant-supported monolithic zirconia crowns (MZCs) cemented to hybrid abutments using AI was presented by Lerner et al. [37]. Combining AI with CAD software made the crown construction process more effective, which decreased production time, expenses, and faults (Figure 4). A three-year survival rate of 99.0% and a success rate of 91.3% were reported in a retrospective analysis of 106 implant-supported MZCs. Furthermore, it can be challenging to identify implant systems if the dentist is unfamiliar with them. Takahashi et al. [38] investigated this by identifying implant systems from panoramic radiographs using DL-based object detection software. According to scientists, this AI-powered strategy has a high possibility of helping patients and dentists better handle implant-related problems (Figure 4) [39].

Advantages of Artificial Intelligence

**Figure 5:**
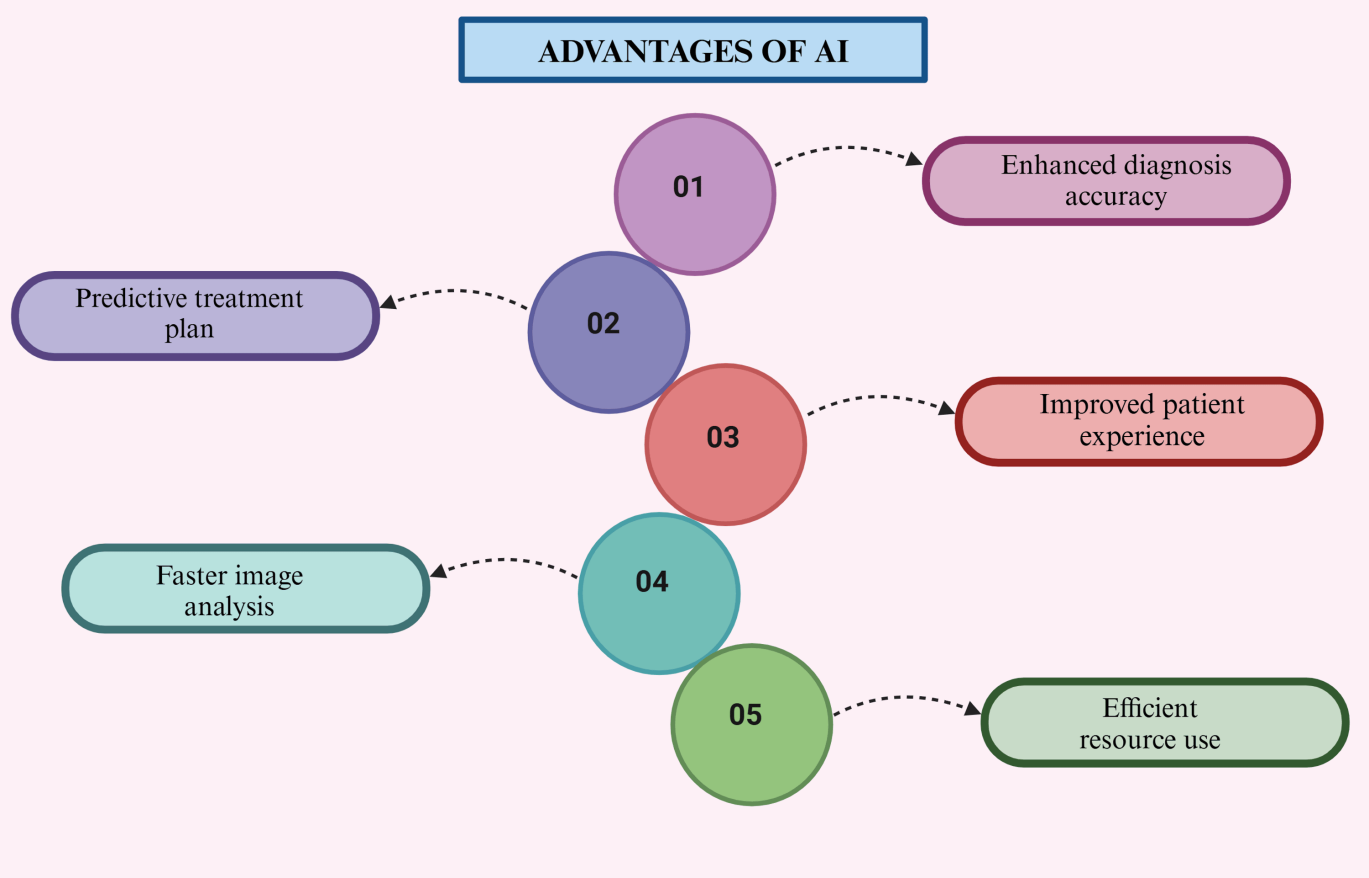
Diagram illustrating the advantages of AI. Note: This figure was drawn using the premium version of BioRender [7] (https://BioRender.com/), accessed on March 9, 2025, which has the agreement license number US280265RW. Illustration credit: Maitri Patel AI, artificial intelligence

AI is transforming periodontology by improving diagnosis, treatment, and patient care. It enables early detection of periodontal diseases through clinical and radiographic analysis, allowing timely intervention. ML helps create personalized treatment plans based on patient-specific factors, while AI-driven radiographic analysis ensures accurate identification of disease indicators. In surgery, AI-powered robotics enhance precision, reducing errors in procedures such as flap surgeries and dental implants. AI also supports patient monitoring via mobile apps, alerts for disease progression, and decision-making through clinical support systems. Additionally, it streamlines administrative tasks, saving time and costs. These advancements make AI a game-changer in periodontology, enhancing accuracy, efficiency, and patient outcomes (Figure 5) [40].

Disadvantage of Artificial Intelligence

**Figure 6:**
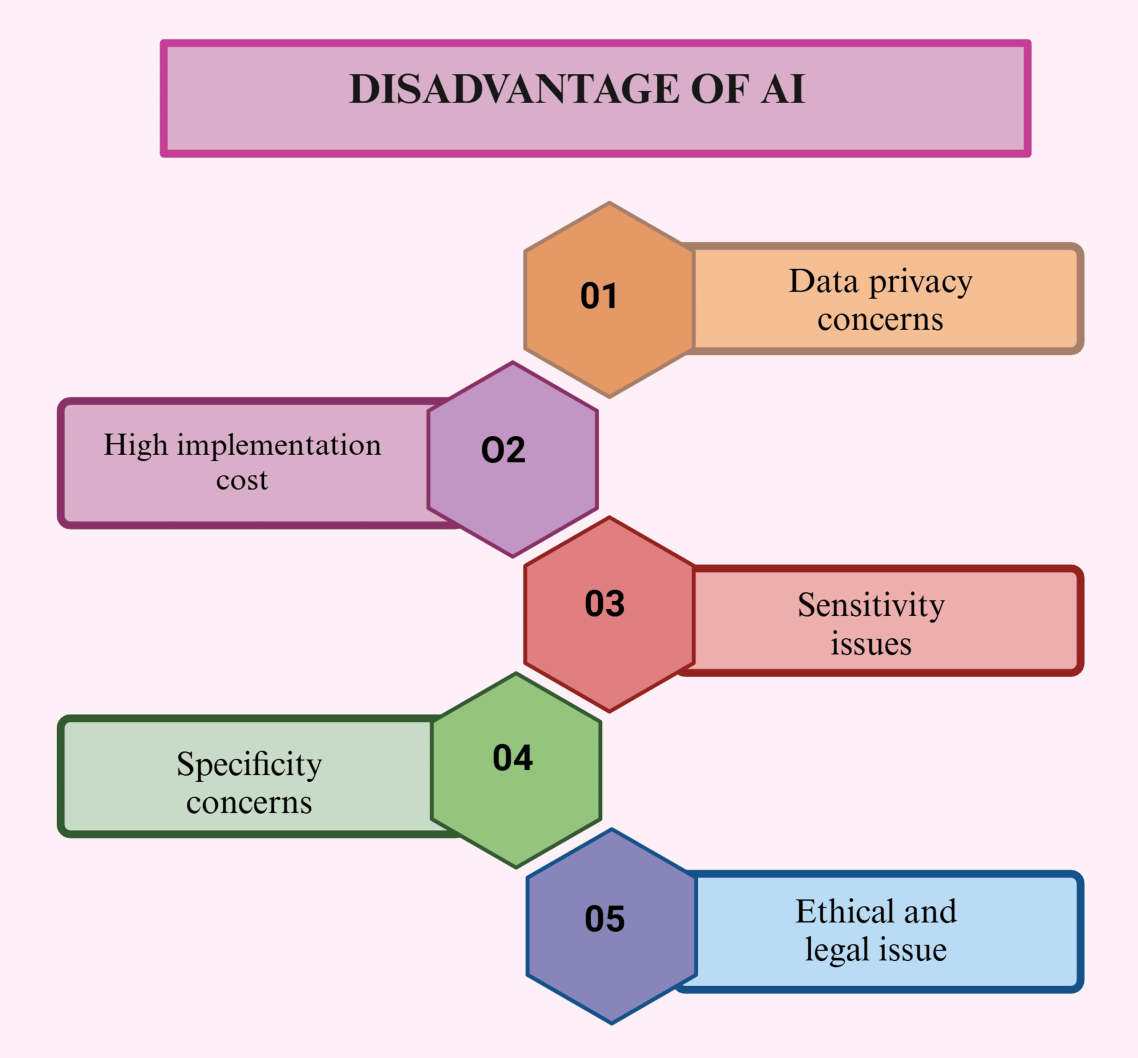
Diagram showing the disadvantage of AI. Note: This figure was drawn using the premium version of BioRender [7] (https://BioRender.com/), accessed on March 9, 2025, which has the agreement license number RM2802E9B9. Illustration credit: Maitri Patel AI, artificial intelligence

While AI holds significant promise in transforming periodontics, its integration comes with various challenges and ethical considerations. Key concerns include patient data privacy, potential algorithm biases, and the necessity for ongoing validation and refinement of AI systems. To fully and responsibly leverage AI's potential, it is essential to prioritize transparency, uphold ethical standards, and ensure alignment with established clinical guidelines [41]. As AI becomes more integrated into healthcare, ensuring ethical use and regulatory compliance is essential. Ethical considerations include patient privacy, obtaining informed consent for AI-driven procedures, maintaining algorithmic decision-making transparency, and addressing AI systems biases. Regulatory bodies and professional organizations are vital in setting guidelines and standards to govern AI's ethical implementation in periodontics. Ongoing monitoring, validation, and auditing of AI systems are crucial to uphold their accuracy, reliability, and ethical integrity. Periodontists and dental professionals must stay informed and proactive in adapting to evolving best practices in AI applications within their field (Figure 6) [42].

Advancing Role of Artificial Intelligence in Dentistry

AI technologies in dentistry can support future advancements in patient-centered, individualized treatment [43]. These technologies can also be crucial in integrating patient data management, healthcare applications, and services. Despite the obstacles, the use of AI in dentistry appears to have a bright future. Research and development are still being conducted to enhance AI algorithms and increase their use in dentistry. For instance, scientists are looking at using AI in orthodontics to direct tooth movement and forecast treatment results.

**Figure 7:**
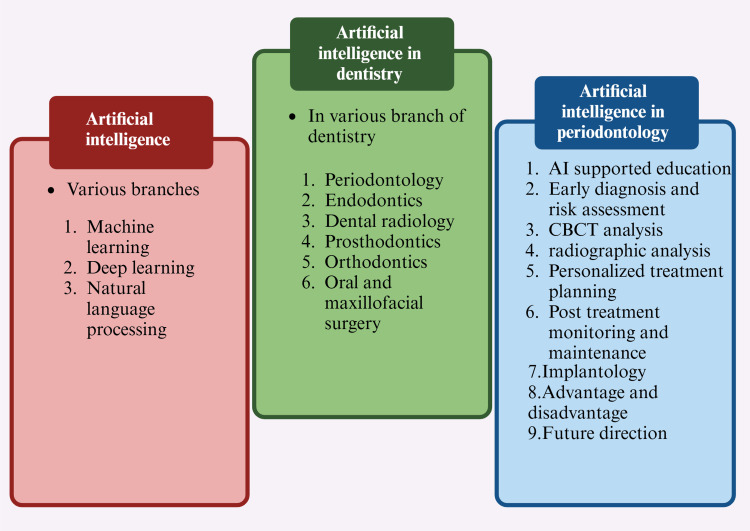
Schematic representation of the fundamental findings of this paper. Note: This figure was drawn using the premium version of BioRender [7] (https://BioRender.com/), accessed on March 9, 2025, which has the agreement license number JI2802K1XI. Illustration credit: Maitri Patel CBCT, cone beam computed tomography

Furthermore, AI-driven robotics is being developed to increase the accuracy and precision of dental procedures [44]. These developments have the potential to completely transform dentistry and improve patient and practitioner outcomes [45]. Figure 7 sketches the principal findings of this narrative review.

Health Insurance Portability and Accountability Act (HIPPA)

Compliance with HIPAA:* *HIPAA establishes standards to protect sensitive patient data. Organizations handling protected health information (PHI) must implement security measures to ensure compliance. HIPAA has three key components: the Privacy Rule, regulating PHI use and granting patient rights; the Security Rule, requiring administrative, physical, and technical safeguards; and the Breach Notification Rule, mandating notifications after a data breach. Compliance involves conducting risk assessments, enforcing policies, providing employee training, and maintaining detailed records. Continuous monitoring and updates are essential to ensure ongoing compliance and protect patient information [46].

How AI will manage and prevent patient data breaches*: *AI plays a crucial role in preventing patient data breaches in healthcare by enhancing security systems and detecting threats. It monitors network activity in real-time to identify anomalies using ML to detect malicious behavior and prevent unauthorized access. AI ensures data encryption, enforces secure access through biometric or multi-factor authentication, and performs behavioral analysis to flag suspicious activities. In a breach, AI triggers automated incident response protocols to contain the threat. Additionally, it anonymizes patient data, ensures regulatory compliance, and conducts predictive analysis to detect potential risks. AI-powered simulations also enhance employee training, strengthen cybersecurity, and protect patient information [47]. Future studies should establish a gold standard for evaluating AI performance across healthcare contexts using independent tests with representative sample data. A standardized framework is essential to help clinicians interpret AI metrics and assess its impact on patient safety, reliability, and uncertainty. Data standardization and integration across healthcare systems are crucial to improving AI performance. Medical education should also incorporate AI training to ensure providers can effectively interpret and apply AI insights in clinical decision-making [48].

Limitations of this study

While AI holds great potential in periodontology, several limitations hinder its full integration. A key issue is limited clinical validation, as most studies rely on small-scale trials rather than large-scale clinical testing. Data quality and bias also affect AI accuracy, reducing its reliability in diverse patient populations. The lack of standardization in AI methodologies leads to inconsistencies, while ethical and legal concerns arise regarding patient privacy, consent, and liability. Moreover, technological barriers and the need for professional training limit accessibility, emphasizing that AI should complement, not replace, clinical expertise.

Future research recommendation

Future research in AI for periodontology should focus on large-scale clinical trials to validate AI models through multicenter studies, ensuring their effectiveness in real-world applications. Enhancing data diversity and quality by creating comprehensive datasets will improve AI's accuracy and applicability in periodontal diagnosis and treatment [49]. Establishing standardized AI models with universal guidelines is essential for ensuring consistency and reliability across different clinical settings. Research should also address ethical and regulatory concerns, focusing on data privacy, consent, and legal implications. Additionally, exploring AI's integration with clinical decision-making will help optimize its role as a supportive tool rather than replacing dentists [50]. Lastly, evaluating cost-effectiveness and accessibility will determine AI's feasibility in widespread periodontal care.

## Conclusions

AI is transforming the field of dentistry by enhancing diagnostic accuracy, decision-making, treatment planning, and disease prognosis. When trained with objective data and optimized algorithms, AI can significantly reduce the workload of dental professionals while improving efficiency. However, regulatory approval, integration into healthcare systems, standardization, training, and sustainable funding are essential for widespread implementation. Despite its advantages, AI cannot replace clinicians, but it is a valuable support tool. Periodontists and other dental professionals will continue to play a crucial role, utilizing their human skills to provide compassionate, patient-centered care.
